# Neck and capsid architecture of the robust *Agrobacterium* phage Milano

**DOI:** 10.1038/s42003-023-05292-1

**Published:** 2023-09-08

**Authors:** Ravi R. Sonani, Nathaniel C. Esteves, Abigail A. Horton, Rebecca J. Kelly, Amanda L. Sebastian, Fengbin Wang, Mark A. B. Kreutzberger, Petr G. Leiman, Birgit E. Scharf, Edward H. Egelman

**Affiliations:** 1 Department of Biochemistry and Molecular Genetics, University of Virginia School of Medicine, Charlottesville, VA 22903 USA; 2 Department of Biological Sciences, Virginia Tech, Blacksburg, VA 24061 USA; 3 Department of Biochemistry and Molecular Biology, University of Texas Medical Branch, Galveston, TX 77555 USA; 4 Present Address: Department of Biochemistry and Molecular Genetics, University of Alabama at Birmingham, Birmingham, AL 35233 USA

**Keywords:** Cryoelectron microscopy, Supramolecular assembly

## Abstract

Large gaps exist in our understanding of how bacteriophages, the most abundant biological entities on Earth, assemble and function. The structure of the “neck” region, where the DNA-filled capsid is connected to the host-recognizing tail remains poorly understood. We describe cryo-EM structures of the neck, the neck-capsid and neck-tail junctions, and capsid of the *Agrobacterium* phage Milano. The Milano neck 1 protein connects the 12-fold symmetrical neck to a 5-fold vertex of the icosahedral capsid. Comparison of Milano neck 1 homologs leads to four proposed classes, likely evolved from the simplest one in siphophages to more complex ones in myo- and podophages. Milano neck is surrounded by the atypical collar, which covalently crosslinks the tail sheath to neck 1. The Milano capsid is decorated with three types of proteins, a minor capsid protein (mCP) and two linking proteins crosslinking the mCP to the major capsid protein. The extensive network of disulfide bonds within and between neck, collar, capsid and tail provides an exceptional structural stability to Milano.

## Abstract (web summary)

Cryo-EM structures of the neck, neck-capsid and neck-tail junctions, and the capsid of the phage Milano reveal a unique collar structure and an extensive network of disulfide bonds providing structural stability to the phage.

## Introduction

Bacteriophages have regained attention in the last few years^1–3^ due to their potential in combating bacterial infections and overcoming antibiotic resistance. *Caudovirales*, the taxonomic order for the tailed double-stranded DNA bacteriophages, is characterized by the presence of a tail—a host cell attachment organelle that is connected to the genome-containing spherical or elongated capsid via a neck^4^. *Caudovirales* is sub-classified into three families: Myoviridae, which carry a long contractile tail; Siphoviridae that possess a long non-contractile tail; and Podoviridae with a very short non-contractile tail^5^. The tubular part of the tail is formed by helical polymerization of a tube protein in siphophages and an additional sheath protein in myophages^6^.

The neck connects the tail to the unique vertex of the capsid that is occupied by a dodecameric portal protein^7^. The neck and capsid contain a varying number of proteins that display a substantial structural diversity. For example, the neck may or may not be surrounded by collar-like structures^8–15^. Besides serving as a symmetry adaptor between a capsid and tail, the neck controls the release of phage DNA into the host cell during infection^8,16,17^. The tail to capsid junction involves a symmetry mismatch that is bridged by the neck, and is therefore crucial for the assembly of tailed phage. The mismatch is overcome by a head-proximal component of the neck establishing unique contacts to the icosahedral capsid. The structure of the neck alone has been studied for several phages^8–18^, and two studies have examined the symmetry mismatch at the neck-capsid interface^8,18^, which were distinctly different between the podophage Sf6 and myophage T4.

The fundamental skeleton of the capsid shell in all *Caudovirales* is formed by the icosahedral arrangement of hexamers and pentamers of the major capsid protein (MCP) having a conserved HK97-fold^19–21^. This capsid shell can be “decorated” by additional domains of the MCP or minor capsid proteins (mCPs) that play an important role in capsid stability and may participate in host recognition during infection.

Bacteriophage Milano^22^ is a myophage that infects *Agrobacterium tumefaciens*, a plant pathogen that is being widely used in the genetic manipulation of plants. Milano requires an actively rotating flagellum on the surface of the bacterial host to which it binds to initiate an infection. Milano’s genome (68 kb) has only recently been sequenced^22^. Its predicted proteome contains 1.4% cysteine residues, which is significantly higher than the cysteine content in other bacteriophages (e.g., 0.9% in T4 and 0.7% in A511 phage). Remarkably, these cysteines are even more prevalent (2.4%) in predicted structural proteins.

Here, we report the cryo-EM structures of various regions of the neck, including the neck-capsid and neck-tail junctions, as well as the capsid of Milano. We describe how the neck 1 protomers undergo structural polymorphism to connect the 12-fold neck with the 5-fold capsid vertex. Our results reveal the exceptional structure of the Milano collar, departing from the typical triple-helical coiled-coil fold observed in other phages, and how it crosslinks the tail sheath to the neck. The Milano capsid is decorated with a mCP and two linking proteins (LP1 and LP2), crosslinking the MCP to the mCP. Our structures further reveal an extensive network of disulfide bonds within and between neck, collar, capsid and tail, and we show how these bonds provide structural robustness to the phage.

## Results

### Cryo-EM reconstructions and structures of Milano neck region

**Fig. 1:**
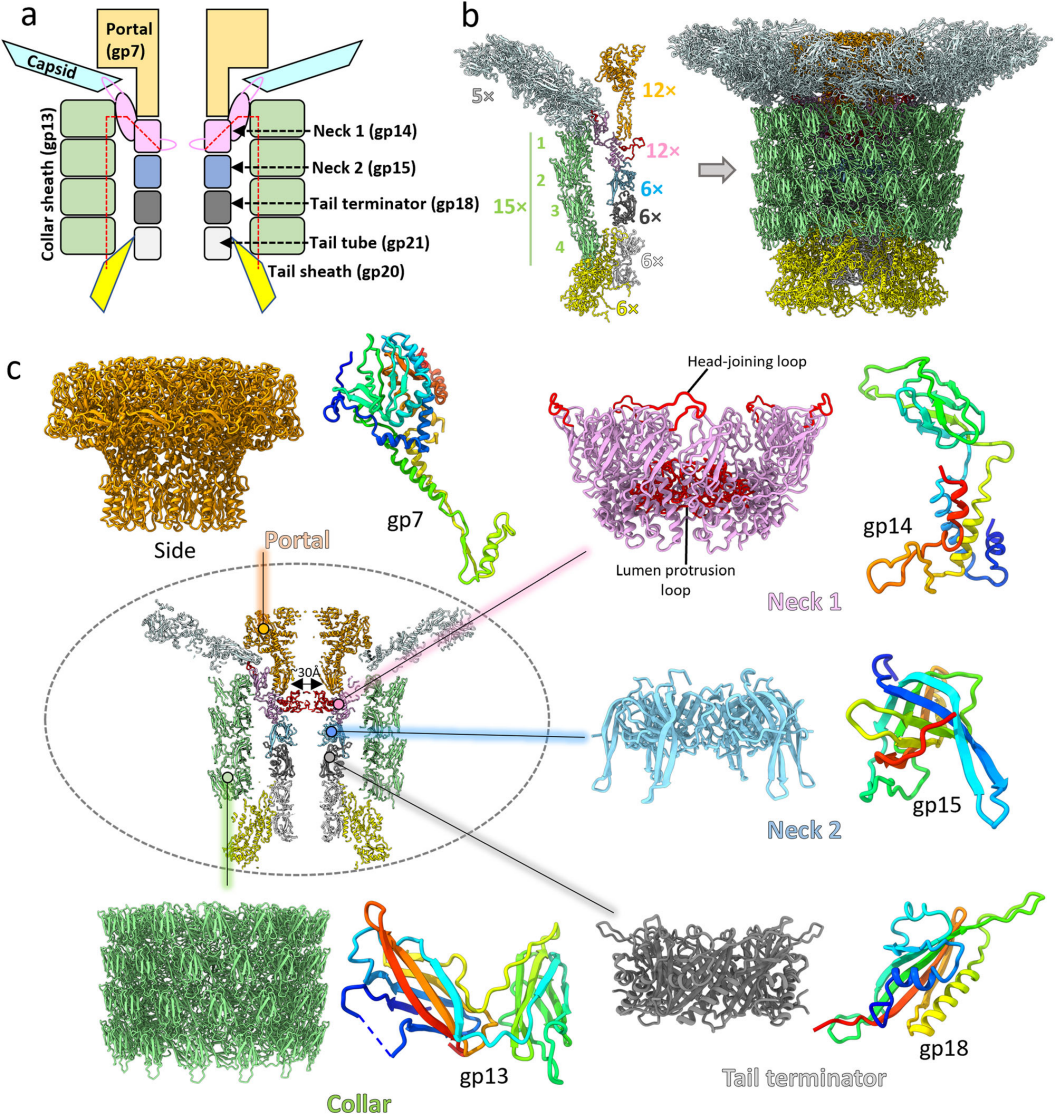
Cryo-EM structure of Milano neck. **a** Schematic of structural organization of Milano neck. The covalent connection between the neck 1 lumen protrusion loop and the tail sheath through the collar is indicated by the red dashed line. **b** Milano consists of stacked protein rings having different symmetries. The portal, neck 1, neck 2/tail terminator and collar has C12, pseudo-C12, C6 and C15 symmetry, respectively. Tail terminator connects the neck to the C6 tail tube and tail sheath. The C12 portal ring is embedded into the capsid at the 5-fold portal vertex. **c** Enlarged view of portal, neck 1, neck 2, collar and tail terminator rings with their component subunits colored blue to red, N- to C-terminal.

Six cryo-EM volumes have been reconstructed for different parts of the Milano neck by imposing different symmetries (Fig. 1, Table 1). The 15-fold, 12-fold and 3-fold symmetrized maps of the neck were used to build atomic models of the collar, portal, and neck devoid of capsid (portal + neck 1 + neck 2 + tail terminator + collar), respectively. The 5-fold symmetrized map was used to build the model of the neck-capsid junction — the neck 1 and collar interacting with the 5-fold capsid vertex. A 3-fold symmetrized map focused at the neck-tail junction was used to understand the interactions between the collar and the tail. Finally, a no-symmetry-imposed map was used to generate an atomic model of the complete neck.

**Table 1:** Cryo-EM data collection and refinement statistics of Milano Neck reconstructions and models.

	Portal	Collar	Neck C3	Neck-Capsid junction	Neck-tail junction	Neck C1	Capsid
PDB entry	8FWB	8FWC	8FWE	8FWG	8FWM	8FXR	8FXP
EMBD entry	29500	29501	29503	29504	29512	29541	29540
Voltage (kV)	300	300	300	300	300	300	300
Magnification (x)	81,000	81,000	81,000	81,000	81,000	81,000	81,000
Electron exposure (e-/ Å^2^)	50	50	50	50	50	50	50
Pixel size (Å/pixel)	1.08	1.08	1.08	1.08	1.08	1.08	1.44
Particle images (no.)	10,216	10,216	10,216	10,083	10,216	10,083	15,740
Symmetry imposed							
Point group	C12	C15	C3	C5	C3	C1	C5
Map global resolution (Å)							
Map:map FSC (0.143)	3.1 Å	3.0 Å	3.5 Å	3.5 Å	3.5 Å	4.5 Å	4.0 Å
Model:map FSC (0.5)	3.4 Å	3.1 Å	3.6 Å	3.6 Å	3.6 Å	4.7 Å	4.5 Å
Refinement and model validation							
Clash score	11.58	7.24	12.62	10.48	13.63	12.14	17.83
R.M.S. deviations							
Bond Length (Å)	0.005	0.009	0.011	0.005	0.004	0.003	0.011
Bond Angle (°)	0.819	0.993	0.865	0.733	0.844	0.680	0.781
Ramachandran plot (%)							
Outliers	0.00	0.00	0.18	0.10	0.35	0.15	0.41
Allowed	12.82	10.33	11.50	12.06	13.63	10.09	13.71
Favoured	87.18	89.67	88.32	87.84	86.01	89.76	85.88

The inner part of the Milano neck complex is similar to that found in T4 and *Vibrio* XM1 bacteriophages^10,11^ (Fig. 1, Supplementary Figs.  S1, S2). Namely, the dodecameric portal ring (12 × gp7), which is embedded into the capsid shell, connects sequentially to the dodecameric neck 1 ring (12 × gp14), hexameric neck 2 ring (6 × gp15) and hexameric tail terminator ring (6 × gp18). The latter caps the tail tube and interacts with the sheath (see below). Besides these conserved common elements, the Milano neck complex possesses a unique, external, sheath-like structure surrounding the neck and consisting of four pentadecameric rings of gp13 (15 × gp13) that we name the collar.

As observed in the structure of R-type pyocin^23^, the tail terminator, while connecting the neck to the tail tube, buries its C-terminal arm in the handshake domain of tail sheath protomers belonging to the top ring^23^ (Supplementary Fig. S3). The arm forms a β-strand that augments the β-sheet of the topmost sheath subunit in a manner resembling interactions between sheath subunits elsewhere in the sheath. The structures of portal, neck 2, and tail terminator rings of Milano show considerable similarity to their counterparts in other bacteriophages at both protomer and oligomeric levels (Supplementary Fig. S2)^10,11,16,18,24–28^. We thus focus on the unique structural features of the Milano neck, the neck 1 and collar.

### Milano neck 1 proteins are polymorphic to bridge the neck to the capsid

**Fig. 2:**
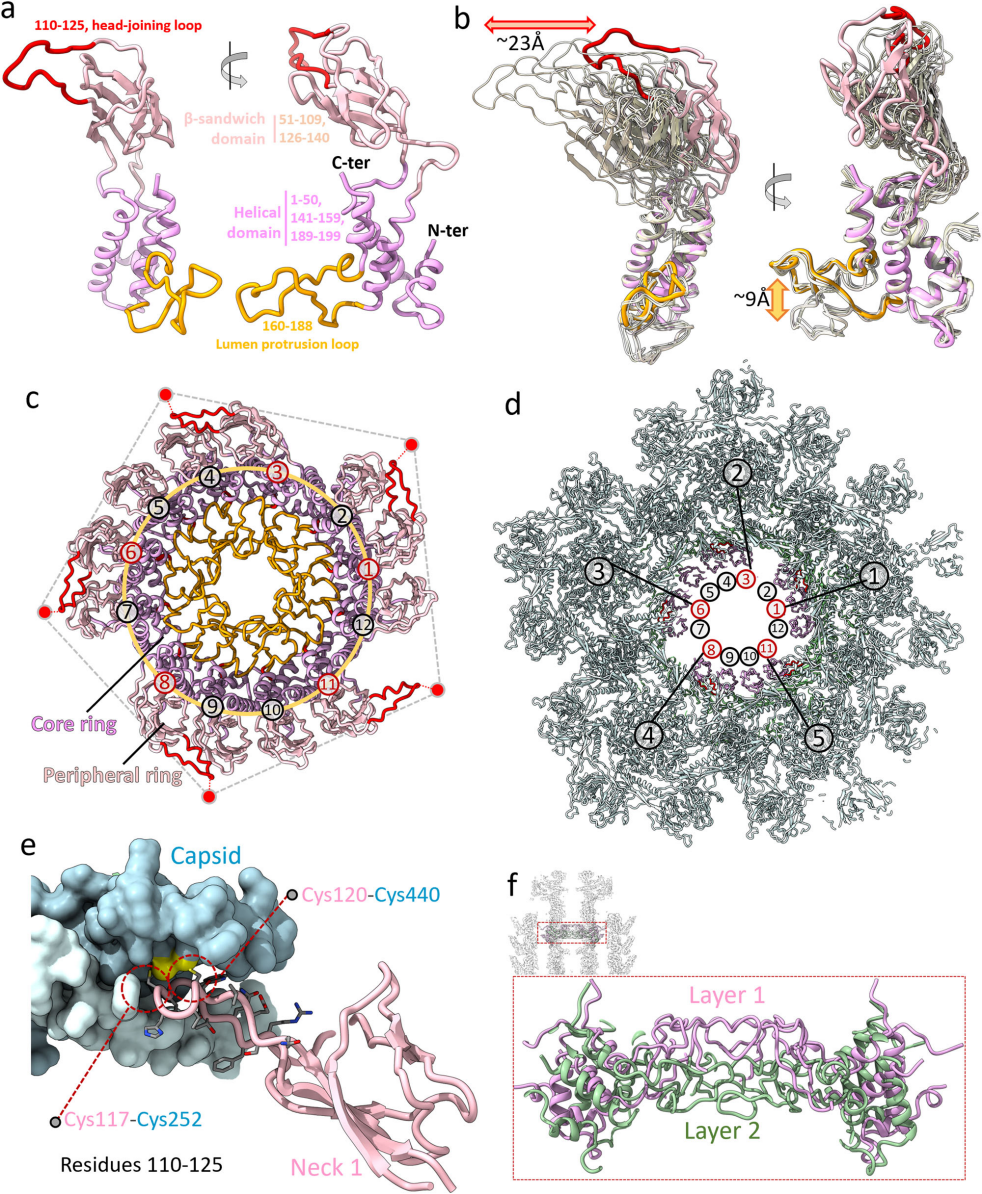
Structural organization of neck 1. **a** Structure of neck 1 protein, gp14. The helical domain, β-sandwich domain, head joining (residues, 110–125) and lumen protrusion loops (residues 160–188) are colored dark pink, light pink, red and orange, respectively. **b** Superimposed view of all 12 protomers of gp14 in the Milano neck 1 ring, aligned at their helical-domain. The different conformations of β-sandwich domain and dual conformations of lumen protrusion loop are indicated by red and orange double-headed arrows, respectively. **c** Top view of neck 1 assembly in Milano. Color scheme is same as Fig. 2a. The β-sandwich domains are clustered asymmetrically in groups of three, four and five. The head joining loop of five protomers (n, n + 2, n + 5, n + 7, n + 10, CCW direction) are connected to MCPs at the 5-fold portal vertex, indicated by red circles. **d** Same view of Fig. 2c with capsid (grey), showing five out of twelve gp14 protomers (n, n + 2, n + 5, n + 7, n + 10, CCW direction) deviating from C12 symmetry to connect with the 5-fold capsid. **e** Interaction of the head joining loop of gp14 with MCP (gp9). The gp14 and gp9 subunits are shown as pink ribbon and cyan surface models, respectively. Cysteines involved in disulfide bonds are colored yellow and indicated by red dashed circles. **f** The lumen protrusion loop of gp14, by adopting a dual conformation, creates a two layered diaphragm-like structure in the lumen of the Milano neck. Two different layers created by different conformations are shown in pink and green.

Milano neck 1 protomer (gp14) consists of two main domains (Fig. 2): an α-helical domain (residues 1–50, 141–159 and 189–199) containing four helices, a β-sandwich domain (residues 51–109 and 126–140) containing six anti-parallel β-strands, and two loops: a head joining loop (residues 110–125), and a lumen protrusion loop (residues 160–188) (Fig. 2a). The β-sandwich domain of Milano neck 1 is absent in its homologs in XM1, HK97, SPP1, P22, Sf6 and Mu, but present in T4 and T7 phages (Fig. 2b, Supplementary Fig. S2A)^11,16,26,29,30^.

In the dodecameric neck 1 assembly, the α-helical and β-sandwich domains of gp14 form the core and peripheral ring, respectively (Figs. 1c, 2c). The core ring connects the neck 2 ring to the portal. The lumen protrusion loops form a diaphragm-like structure that constricts the channel spanning all proteins of the neck and the portal protein. These loops decrease the diameter of the neck lumen from ~30 Å to ~18 Å. The loop is formed by mostly polar residues, including a positively charged Arg167, which gives this diaphragm an overall positive charge (Supplementary Figs. S1C, S4). There are two alternating conformations of the lumen protrusion loop, with six tilted up and six tilted down. The RMSD between these two conformations of the loop is 9.7 Å (Fig. 2b).

The peripheral ring of neck 1 breaks the C12 symmetry of the core and connects the neck to the capsid. The 12 ring-forming gp14 protomers exhibit structural polymorphism. Their β-sandwich domains adopt different orientations in each protomer and can be described as 12 steps in a continuous tilting relative to the α-helical domain (Fig. 2b). When the α-helical domains of the 12 gp14 subunits are superimposed, they display an RMSD of 2.6 Å, while the β-sandwich domains have a varying orientation with the maximal distance between their distal parts reaching 24 Å. The β-sandwich domains are clustered in three groups with 3, 4, and 5 members each (Fig. 2c, d) creating a 12-to-5-fold symmetry adaptor assembly to bridge neck-capsid symmetry mismatch.

Five out of 12 gp14 β-sandwich domains (n, n + 2, n + 5, n + 7, n + 10, CCW direction looking from the capsid side) display pseudo-5-fold symmetry (Fig. 2c, d). These domains are covalently linked to the MCP (gp9) by two disulfide bonds, ^gp14^Cys117– ^gp9^Cys252 and ^gp14^Cys120– ^gp9^Cys440, which emanate from a head joining loop of the β-sandwich domains (Fig. 2e). The head joining loops of the remaining seven gp14 protomers are not resolved in our maps and thus likely to be disordered. The β-sandwich domains form a positively charged “bowl”, which houses a negatively charged portal clip domain region^31^ thus strengthening the connection between the portal and the neck tube (Supplementary Fig. S1C).

In summary, in the gp14 neck 1 dodecamer, the α-helical domain connects the portal to neck 1 ring smoothly while maintaining C12 symmetry, while its β-sandwich domain binds to the five-fold symmetric capsid vertex by adopting 12 different conformations. Electrostatics appears to play a role in the interaction of the β-sandwich domain with the portal clip region. Finally, lumen protrusion loops form a two layered diaphragm-like structure in the lumen of the neck by adopting two alternating conformations (Fig. 2f).

### The collar crosslinks the tail sheath to neck 1

The Milano neck is covered by a sheath-like structure in place of the collar/whiskers (gp *wac*) of T4^10^, collar spikes (gp40) of XM1^11^, and pre-neck appendage (equivalent to collar, gp12) of phi29^32^ phages. No protruding fibrous elements such as whiskers/spikes/appendages emanate from the Milano collar.

**Fig. 3:**
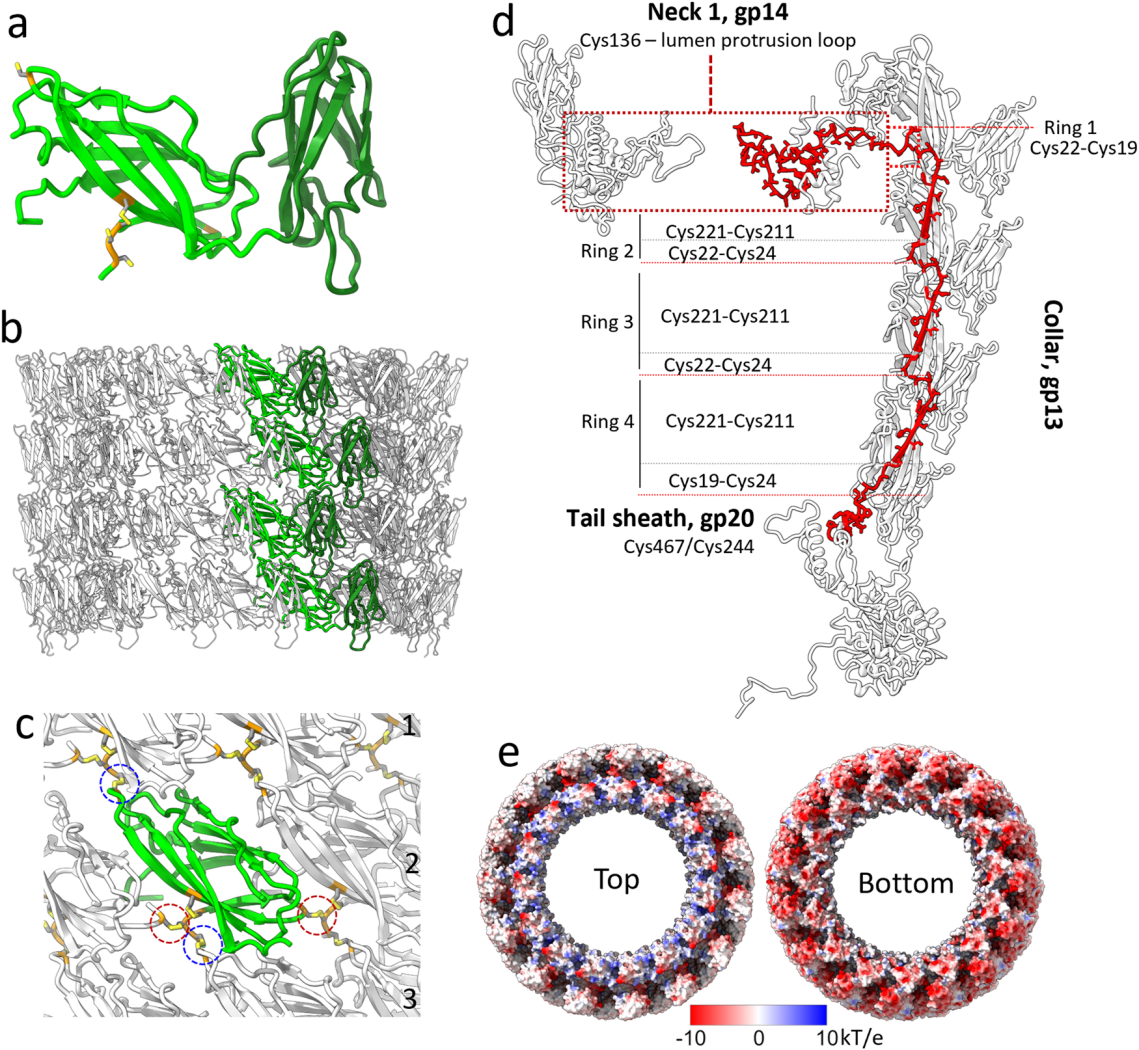
Structural organization of the collar. The structure of (**a**) collar protomer gp13 and (**b**) assembly. The W (wall)- and D (decorating) domains of gp13 are colored light and dark green, respectively. Cysteines involved in disulfide bonds are colored yellow. **c** Disulfide bond network between collar subunits. Each gp13 protomer makes four disulfide bonds, one with each neighbor within the same ring (Red circles, Cys23-Cys229), and one with each neighbor related by left-handed 15-start helices in an adjacent ring (Blue circles, Cys22-Cys211). **d** The collar forms a covalent structural link between the tail sheath and the neck 1 lumen protrusion loop: [sheath ^gp20^Cys467/Cys244] → [collar ^gp13^Cys19-Cys24 _ring4_ → ^gp13^Cys221-Cys211_ring4_ → ^gp13^Cys22-Cys24_ring3_ → ^gp13^Cys221-Cys211_ring3_ → ^gp13^Cys22-Cys24_ring2_ → ^gp13^Cys221-Cys211_ring2_ → ^gp13^Cys22-Cys19_ring1_] → [neck 1 ^gp14^Cys136—lumen protrusion loop, 160-188]. **e** Electrostatic potential of top and bottom surfaces of gp13 pentadecameric rings.

The Milano collar is made up of four rings of gp13 pentadecamers (15 × gp13). Since this pentadecamer has both the C5 symmetry of the capsid and a C3 symmetry (present in the C6 symmetry of the tail), it is resolved in both the C3 and C5 symmetric reconstructions of the neck (Fig. 1). Interestingly, Milano gp13 is devoid of α-helices, which are found in collar proteins of T4, XM1 and phi29 phages with many of these helices forming triple helical coiled coils^10,11,32^ (Supplementary Fig. S5). The gp13 subunit contains two β-strand-rich jellyroll-like domains. Domain 1 (residues 1–69, 167–230) forms the wall of the collar, and we thus named it the wall domain (W domain), whereas domain 2 (residues 70–166) decorates the collar wall, and we named it the decorating domain (D domain) (Fig. 3a, b). DALI search results suggest that the D domain of gp13 is structurally similar to glycan-interacting and cell surface binding proteins (Supplementary Table S1).

Four collar rings are stacked and stabilized by complementary electrostatic charges on the interacting surfaces of their W domains (Fig. 3e). The W domain of each gp13 protomer is covalently linked to its neighboring subunits by four disulfide bonds, one with each neighbor within the same ring (Cys23-Cys229) and one with each neighbor along the left-handed 12-start helices (Cys22-Cys211) (Fig. 3c). The collar connects the neck 1 with the uppermost layer of the sheath without interacting with the neck components in its midsection. Namely, the head-proximal end of the collar interacts with the β-sandwich domain of neck 1 (gp14), while the tail-proximal end of the collar interacts with the tail sheath (gp20) (Figs. 1a and 3d) (Table 2). Every third (n, n + 3, n + 6, n + 9, n + 12) collar subunit is attached to the neck 1 subunits (n, n + 2, n + 5, n + 7, n + 10, CCW direction looking from the capsid side) through the disulfide bond ^gp14^Cys136—^gp13^Cys19, linking the collar to neck 1 by five disulfide bonds. It is possible that other collar subunits might be making additional disulfide bonds not resolved in our map.

**Table 2:** Inter-chain disulfide bonds within and between sub-structures of Milano neck.

**Within sub-structure**		
	**Residue**	**Bonded To**
Collar	gp13, Cys22	gp13, Cys211
	gp13, Cys23	gp13, Cys229
Capsid	MCP, gp9, Cys302	MCP, gp9, Cys455
	MCP, gp9, Cys186	MCP, gp9, Cys456 (intrachain)
	MCP, gp9, Cys190	MCP, gp9, Cys385 (intrachain)
	MCP, gp9, Cys223	MCP, gp10, Cys105
	MCP, gp9, Cys252	MCP, gp10, Cys74
	mCP, gp10, Cys99	LP1, gp16, Cys3
	mCP, gp10, Cys60	LP1, gp16, Cys7
	mCP, gp10, Cys99	LP2, gp128, Cys6
	mCP, gp10, Cys60	LP2, gp16, Cys10
	LP2, gp16, Cys23	LP2, gp128, Cys26
	LP1, gp16, Cys20	LP2, gp128, Cys26
**Between sub-structures**		
Collar—Neck 1	Collar gp13, Cys19	Neck 1, gp14, Cys136
Tube terminator—Tail tube	Tube terminator, gp18, Cys156	Tail tube, gp21, Cys3
Collar—Tail sheath	Collar, gp13, Cys19	Tail sheath, gp20, Cys467
	Collar, gp13, Cys23	Tail sheath, gp20, Cys244
Neck 1—Capsid	Neck 1, gp14, Cys117	MCP, gp9, Cys252
	Neck 1, gp14, Cys120	MCP, gp9, Cys440

*MCP* major capsid protein, *mCP* minor capsid protein, *LP1* linking protein 1, *LP2* linking protein 2.

The interaction of the C15-symmetric collar with the C6-symmetric tail sheath is more complex. Five collar subunits (e.g., n, n + 1, n + 2, n + 3, n + 4) comprising the asymmetric unit interact with four tail sheath subunits belonging to the two topmost sheath rings (or layers) that we named Fn, Fn+1, Sn, Sn+1, where F and S denote first and second ring, respectively. The collar subunits n (Cys19), n + 3 (Cys19), and n + 4 (Cys23) form disulfide bonds with tail sheath subunits Sn (Cys467), Sn+1 (Cys467) and Fn+1 (Cys244), respectively, thus linking the collar to the tail sheath by nine (three in each of the three asymmetric units) disulfide bonds (Table 2). Intra- and inter-region disulfide bonds of the collar form a covalent connection between the tail sheath and the neck 1 lumen protrusion loop through the collar, running from the tail sheath as follows: [sheath ^gp20^Cys467/Cys244] → [collar ^gp13^Cys19-Cys24 _ring4_ → ^gp13^Cys221-Cys211_ring4_ → ^gp13^Cys22-Cys24_ring3_ →^gp13^Cys221-Cys211_ring3_ → ^gp13^Cys22-Cys24_ring2_ → ^gp13^Cys221-Cys211_ring2_ → ^gp13^Cys22-Cys19_ring1_] → [neck 1 ^gp14^Cys136—lumen protrusion loop, 160-188] (Fig. 3d).

### An extensive network of disulfide bond provides structural stability

Our atomic models reveal that most of the cysteine residues of Milano structural proteins are involved in disulfide bonds, covalently crosslinking the neck, collar, capsid, and tail. Such an extensive network of disulfide bonds would be expected to strengthen the overall structure of the Milano virion. To test this, we compared the ability of Milano to withstand disintegration by ultrasonic disruption with that of phage T4 and Chi, which contain an average number of cysteines and disulfide bonds.

We found that T4 and Chi fragmented within 10 s under sonication-induced mechanical stress, while Milano particles stayed intact for more than 30 s (Supplementary Fig. S6). In contrast, upon reduction of the disulfide bonds with dithiothreitol (DTT), Milano particles disintegrated within 10 s of sonication similar to T4 and Chi in the absence of DTT (Supplementary Fig. S6). Furthermore, DTT-treated Milano particles disintegrated within 90 s when heated to 60 °C, while untreated Milano was resistant to 60 °C heating for more than 5 min (Supplementary Fig. S6). The loss of structure in the presence of a reducing agent suggests that the disulfide bonds provide extended stability to Milano particles.

Other myophages do not possess such a high percentage of cysteines and resultant disulfide bonds. However, the closely related *Agrobacterium* phages, 7-7-1^33^ and OLIVR4 (Taxon ID: 2723772), exhibit high sequence conservation including cysteine residues in their structural proteins (Supplementary Fig. S7). Therefore, the disulfide bonds and robustness of Milano appear to be correlated with the *Agrobacterium* host. The unique collar and the abundance of disulfide bridges in Milano may be an adaptation to the specific environment associated with *Agrobacterium*.

### The capsid is decorated with a minor capsid protein and linking proteins

**Fig. 4:**
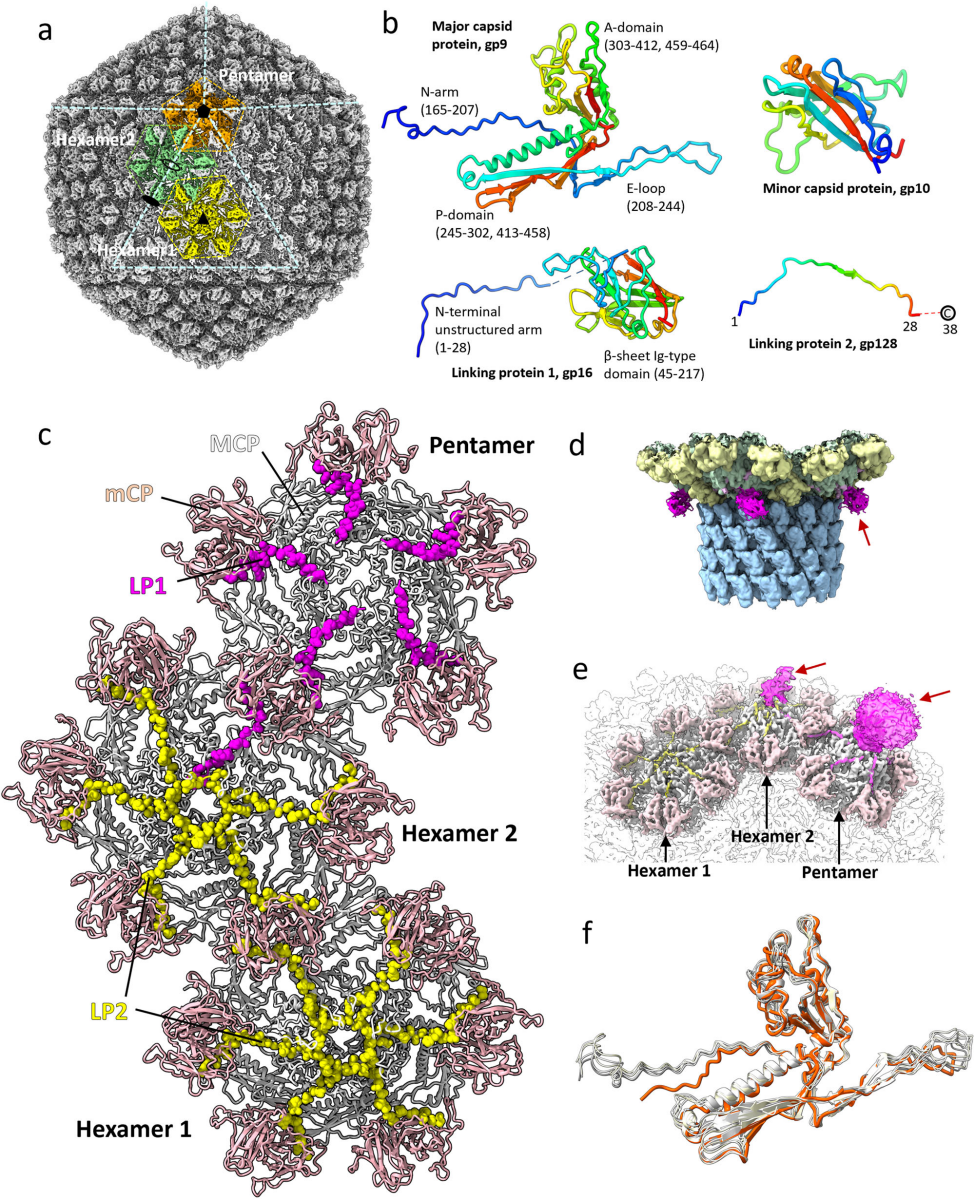
Structure of Milano capsid. **a** Cryo-EM map of the Milano capsid shows that it consists of three building blocks: pentamer (orange), hexamer 1 (yellow) and hexamer 2 (green), each composed of the MCP (gp9). Icosahedral 2-fold, 3-fold and 5-fold symmetry are shown by filled black oval, triangle and pentagon, respectively. The local 2-fold symmetry is shown by an empty oval. **b** Structures of MCP (gp9), mCP (gp10), linking protein 1 (LP1, gp16) and linking protein 2 (LP2, gp128) are shown with N-to C-terminal colored blue-to-red. **c** Structure of pentamer, hexamer 1 and hexamer 2 differing in their decorating proteins. MCP, mCP, LP1 and LP2 are colored white, light pink, pink and yellow, respectively. Pentamer is decorated by five copies of LP2, hexamer1 is decorated by six copies of LP1 and hexamer 2 is decorated by five copies of LP2 and a single LP1. **d** Cryo-EM density corresponding to the β-sheet Ig-type domain of LP1, hanging out of hexamer 2 near the neck (Pink and indicated by red arrow). **e** Cryo-EM density corresponding to the β-sheet Ig-type domain of LP1 at pentamer and hexamer 2 hanging out of the capsid (Pink colored and indicated by red arrows). **f** Different conformations of P-domain, E-loop and N-arm of MCP (gp9) protomers in pentamer (orange) compared to those in hexamer 2 (grey).

The Milano capsid has a T = 9 icosahedral symmetry. It consists of three structural elements that are formed by the MCP (gp9): a pentamer at the icosahedral 5-fold axis, hexamer 1 at the icosahedral 3-fold, and hexamer 2 positioned on a local 2-fold (Fig. 4a). The icosahedral asymmetric unit is formed by a single subunit of the pentamer, two subunits of hexamer 1, and a complete hexamer 2. Hexamer 1 and hexamer 2 differ in their decorating proteins, as described below.

The Milano MCP has a HK97-like fold found in all MCPs of dsDNA tailed phages (Supplementary Fig. S2)^18,27,28^. It consists of an A domain that forms the core, and a P domain, E loop, and N arm that form the peripheral parts of hexamers and pentamers (Fig. 4b). The conformational plasticity in the P domains, E loops, and N arms of the MCP enable the stable construction of quasi-equivalent pentamers and hexamers (Fig. 4f). Hexamers and pentamers are further decorated by additional proteins: mCP (gp10) and two linking proteins (LP)—LP1 (gp16) and LP2 (gp128). The mCP contains a β-strand-rich jellyroll-like domain (Fig. 4b). LP1 is predicted by AlphaFold to have a β-sheet Ig-type domain with an N-terminal unstructured strand. LP2 is a small 38 residue protein with a high degree of sequence similarity to the N-terminal strand of LP1 (Fig. 4b; Supplementary Fig. S8).

A dimer of mCP decorates the external surface of the capsid by cementing the pentamer-hexamer 1 and hexamer 1-hexamer 2 interfaces, where it forms disulfide bonds with the MCPs (^gp9^Cys223—^gp10^Cys105 and ^gp9^Cys252—^gp10^Cys74) (Fig. 4c). The resolved parts of LPs—residues 1-13 of LP1 and 1-10 of LP2—occupy quasi-equivalent positions with an mCP at the periphery of pentamer and hexamers and run towards the centers of the pentamers and hexamers, where they interact with the MCPs (Fig. 4c; Supplementary Fig. S9; Table 2). LP1 and LP2 form all such linkages in the pentamer and hexamer 1, respectively. In hexamer 2, all but one linkage is formed by LP2, while the unique pentamer-proximal link is formed by LP1 (Fig. 4c; Supplementary Fig. S8). Notably, the LP1-mCP and LP2-mCP interaction interfaces also include a disulfide bonds. ^LP1^Cys3/^LP2^Cys6 and ^LP1^Cys7/^LP2^Cys10 form disulfide bond with ^mCP^Cys99 and ^mCP^Cys60, respectively (Table 2; Supplementary Fig. S9).

The C-terminal Ig-type domain of LP1 appears to extend away from the capsid shell without forming much contact with it (Fig. 4d, e). Consequently, the density of the LP1 Ig-type domain is not resolved due to positional disorder. The capsid-decorating Hoc protein of T4, which consists of several Ig-type domains, extends away from the T4 capsid in a similar fashion and the density for these domains is also not resolved^34^. The tethering of Milano’s LP1 to the capsid via an N-terminal arm of LP1 is similar to the way the capsid auxiliary protein gp16 of phage YSD1 binds to its capsid^28^.

While LP2 seems to have only a structural role in strengthening the capsid, the mCP and LP1 may have an additional function. The jellyroll-like domain of mCP and Ig-type domain of LP1 are widespread in phage capsid proteins. The likely function of such proteins and domains is to participate in low-affinity binding to cell surface components (e.g. surface polysaccharides and receptors), which keeps the phage in the vicinity of potential host cells^34–36^. DALI analysis^37^ shows that these proteins are structurally similar to many glycan and receptor binding proteins (Supplementary Table S1), for example, the GlcNAc-binding protein A that mediates attachment of *Vibrio* bacteria to the mammalian intestinal mucin (PDB ID: 2XWX)^38^, the variable domain of T-cell receptor (PDB ID: 1BWM) that binds the MHC I complex antigenic peptide^39^, and the C-terminal domain of a sensor kinase of a *Bacteroides* hybrid two-component system^40^.

## Discussion

Our structural results show that Milano’s neck 1 ring plays a dual structural role: (1) it joins the neck to the capsid and resolves the symmetry mismatch between the two, and (2) it forms a two-layered diaphragm-like structure in the lumen of the neck. The head-to-neck symmetry mismatch has been described for T4^18^ and Sf6^8^ bacteriophages. The N-terminal whisker of the T4 portal protein (gp20) and the C-terminal whisker of the *Shigella* Sf6 head-to-tail adaptor protein (gp7) bridge the neck and head symmetries by conformational differences among its protomers. Our work characterizes how the β-sandwich domains of Milano neck 1 protomers (gp14) adopt different conformations to compensate for the head-to-neck symmetry mismatch and bridge them covalently. While this domain is largely absent in neck 1 homologs of some other bacteriophages, the equivalent proteins of T7 (gp11, PDB id: 6R21)^17^ and T4 (gp13, AlphaFold-predicted, Uniprot# P11110) possess equivalent β-sandwich domains. However, the role of these β-sandwich domains in symmetry adjustment remains to be determined in T4 and T7 phages.

**Fig. 5:**
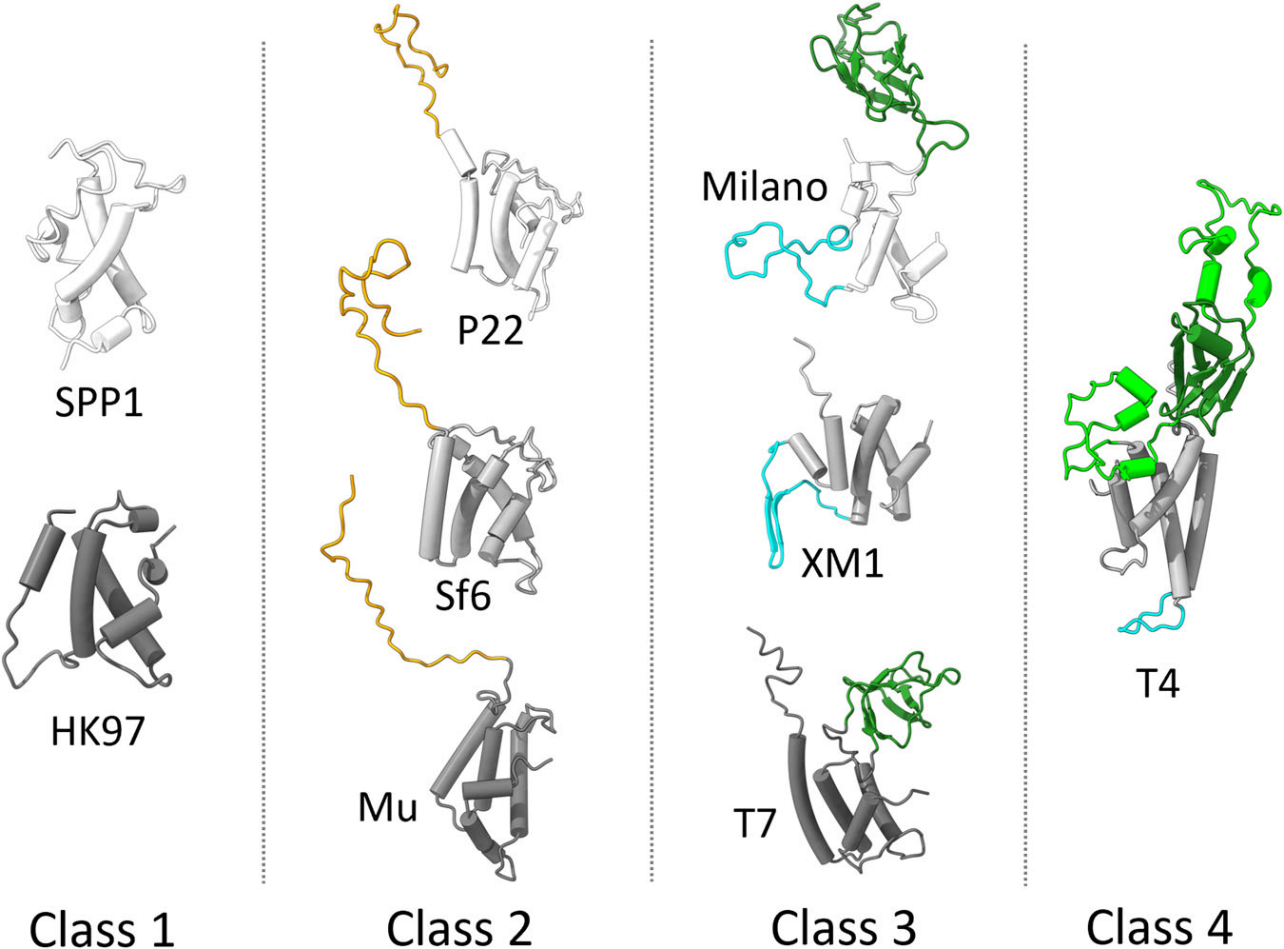
Structural classification of proteins equivalent to Milano neck 1 found in various bacteriophages. The structures for T4 and Mu phages are predicted by Alphafold. The helical domain (white, black, grey), β-sandwich domain (green), C-terminal extension (orange) and stopper loop (cyan) are colored separately.

Structural comparisons of the Milano neck 1 homologs suggest the existence of at least four structural classes of neck 1-like proteins (Fig. 5). Class 1, containing proteins with ~100 residues, represents the simplest form composed of a single helical domain (e.g. gp15 in SPP1 and gp6 in HK97). In Class 2, ~140–160 residue-long proteins possess an additional extended C-terminal strand (e.g. gp4 in P22, gp7 in Sf6, and gp36 in Mu). In Class 3, ~190–210 residue-long proteins possess an additional β-sandwich domain and/or lumen protrusion loop (gp14 in Milano, gp11 in T7, and gp1 in XM1). In Class 4, ~310 residue-long proteins possess two additional α-helical domains protruding out from the β-sandwich domain (gp13 in T4).

These observations suggest an evolutionary pattern of neck 1 protein developing multiple functions. The latter three classes (mostly found in myo- and podophages) appear to have evolved divergently from the same origin as class 1, the simplest structure mostly present in siphophages. Notably, the lumen protrusion loop exists only in myophages, whereas the presence of the β-sandwich domain is spread across myo- and podophages. Therefore, the structural joint between the neck and the portal appears to be the fundamental function of neck 1 in siphophages, whereas the adaptation to genome gating and neck-capsid symmetry bridging may have occurred later in myo- and myo-/podophages, respectively.

The diaphragm-like structure in the Milano neck has two layers formed by two alternating conformations of the neck 1 lumen protrusion loop. It is likely to be flexible in the monomeric state as its AlphaFold-predicted structure exhibits a very different conformation from the two observed experimentally in the cryo-EM map. It has been shown that structural homologs of Milano neck 1 exist as monomers in solution and oligomerize only in the presence of the portal and other neck proteins^29,41^. Two conformations of the neck 1 lumen protrusion loops are also likely to be acquired during neck assembly. Stacks of layer 1 and 2 are stabilized by inter-layer hydrogen bonds between polar residues.

The function of the neck 1 diaphragm structure in the Milano neck remains to be determined. The diaphragm decreases the effective diameter of the neck lumen to <20 Å, which is too narrow for the double-stranded DNA to pass through. Given its polar and positively charged nature, it is likely to interact with the negatively charged DNA. The low-resolution density likely corresponding to the DNA near this diaphragm provides evidence for such an interaction (Supplementary Fig. S10). The diaphragm-like structure must be opened-up or widened for the release of the genome. The lumen protrusion loop of the neck 1 homolog in XM1 phage adopts an orientation parallel to the neck’s central axis and provides a surface for neck 2 interaction^11^. The Milano neck 1 diaphragm might be adopting a similar structure in an open state to facilitate the genome gating.

The lumen of portal, neck 1, and neck 2 possesses a positive electrostatic potential resulting in a positive charge of the neck lumen (Supplementary Fig. S1)—suitable to hold and position the end of the negatively charged linear dsDNA until the genome release. In contrast, the tail terminator ring possesses a negatively charged lumen, repelling the DNA from the wall and thereby lubricating its passage (Supplementary Fig. S1).

The collar of Milano is structurally distinct from that of T4 and XM1 bacteriophage. In both, T4 and XM1, the collar is made up of one ring/disk that surrounds the neck and carries fibers or spikes. In contrast, the Milano collar consists of four rings (4 x gp13_15_) surrounding the whole neck and displaying no fibers or spike-like structures. The T4 collar whiskers (gp *wac*) are important for assembly of tail fibers and control of infection^42^. Based on those findings, XM1 collar spikes (gp40) were proposed to have a role in host sensing and attachment^11^. The biological function of the Milano collar is currently unknown. An elaborate network of disulfide bonds in the collar suggests that they play a role in the overall robustness of Milano particles against mechanical stress (Supplementary Fig. S6), supported by the observation that this stability is lost under reducing conditions. Additionally, the Milano collar connects the sheath to the diaphragm-like structure formed by the neck 1 lumen protrusion loop (Fig. 3d), allowing for the sheath contraction signal to reach to the genome directly, instead of indirectly through the neck proteins, in preparation for genome release during the attachment to the host cell.

Finely, we note that the MCP and mCP are doubly linked: (1) by direct disulfide bonds between MCP and mCP, and (2) through linking proteins LP1 and LP2, which are bound to both MCP and mCP. This likely provides additional robustness to the phage particle.

## Methods

### Cultivation and purification of Milano particles

Bacteriophage Milano stock was prepared using an overlay-plate method. *Agrobacterium tumefaciens* C58 cells were grown in TYC (5 g/l Bacto tryptone, 3 g/l yeast extract, 0.86 g/l CaCl_2_ 2xH_2_O) to an OD_600_ of 0.6. Motility was verified via phase contrast microscopy. Motile cells were infected with Milano phage (10^6^ pfu) and mixed with 0.35% soft agar TYC, layered on 1.5% agar plates and incubated overnight at 30 °C until plaques formed. Phage particles were recovered by overlaying plaque plates with 5 ml TM buffer (50 mM Tris-HCl, pH 7.4 and 10 mM MgSO_4_), and rocking overnight at 4 °C. Phage particles were released from cells by the addition of 1% chloroform and precipitated with 1 M NaCl and 10% PEG8000 under overnight stirring. Phage particles were purified by 10–50% OptiPrep™ linear density gradient ultracentrifugation at 200,000 × *g* for 2 h. Purified phages were extracted and dialyzed against TM buffer in a 10,000 MWCO Thermo Scientific Slide-A-Lyzer® dialysis cassette. Purified phage sample was titered by plaque assay.

### Cryo-electron microscopy

#### Cryo-EM Sample preparation, data collection, and image preprocessing

Holey C-flat carbon grids (1.2/1.3, 400 mesh, copper) were glow-discharged for 30 s in a GATAN Solarus Plasma Cleaner. A 3 µL phage sample in TM buffer was applied to glow-discharged grid, blotted for 3 s and plunge-frozen in liquid ethane (−180 °C) using an EM GP Plunge Freezer (Leica). Cryo-EM data was collected on a 300 keV Titan Krios with a Gatan K3 camera (University of Virginia). Total exposure per movie was ~50 e Å^–2^ with a pixel size of ~1.08 Å. Micrographs were preprocessed by ‘*patch motion correction*’ and ‘*patch CTF estimation*’ jobs in cryoSPARC for motion correction and contrast transfer function (CTF) estimation, respectively^43–45^.

#### Reconstruction of various neck regions

The 3D reconstructions for various neck regions were achieved by single particle analysis (SPA) using cryoSPARC^45^. Neck region of Milano was manually boxed in several micrographs to generate 2D class averages for automatic template-based particle picking by ‘*Template picker*’. Bad picks were removed by iterative ‘*2D-classification*’ and ‘*selection of 2D-classes*’. The ab initio 3-D model was generated by ‘*ab-initio reconstruction*’ job. The ab initio map was further refined by imposing C3 symmetry to the highest possible resolution by ‘*homogenous refinement*’ job. The visual inspection of C3 map suggested different symmetries for different regions of neck. The C3 map was then further refined by imposing/relaxing symmetries (C1, C3, C5, C12 and C15) to generate the highest possible resolution maps for the whole neck assembly, neck devoid of capsid (portal, neck 1, neck 2, tail terminator, tail tube and collar), neck-capsid junction (neck 1, collar and capsid), portal only and collar only. Refinement was performed by iterative cycles of ‘*homogenous refinement*’, ‘*nonuniform homogenous refinement*’ and/or ‘*local CTF refinement*’. The map for the neck-tail junction was refined by ‘*focused refinement*’ with C3 symmetry.

#### Reconstruction of capsid

Choice and selection of Milano capsid particles was done according to a similar procedure used for the neck. Due to the large size of the capsid, the pixel size was down sampled to 1.44 Å before reconstruction. The ab initio model of capsid was generated by ‘*ab-initio reconstruction*’ job and refined by iterative cycles of ‘*homogenous refinement*’ and ‘*CTF refinement*’ jobs imposing icosahedral and C5 symmetries to highest possible resolutions.

The statistics of data collection and processing are detailed in Table 1. The resolution of maps and map-to-map Fourier shell correlation curves generated from cryoSPARC are provided in Supplementary Fig. S11 for all reconstructions.

### Model building

Three-dimensional structures for all 127 proteins of Milano were predicted by AlphaFold^46^. The protein structure models were rigid-body fitted into 3D maps manually and by DeepTracer, as needed^47^. The rigid-body fit models were refined against density map by iterative cycles of automatic refinement using PHENIX^48^ and interactive refinement in Coot^49^. Model building exercise and sequence analysis revealed the error in ORF annotations in the published Milano genome^22^. The density for the N-terminal 56 residues of collar (Uniport# A0A482MGH3) was not present in the map, but instead appeared as a separate protein, for which density had been found in the capsid. Therefore, annotated collar ORF (Uniport# A0A482MGH3) actually starts at Met-57 and its previously annotated residues 1–38 represents another capsid protein (gp128) in a different reading frame that we named linking protein 2 (personal communication with Author of Reference^22^) (Supplementary Fig. S8). The statistics of model building are detailed in Table 1.

### Phage disintegration assay

*Sonication-induced disintegration:* Bacteriophage samples (1 × 10^10^ pfu mL^−1^ in TM buffer) were ultra-sonicated on ice by the XL-2000 series probe ultra-sonicator (MISONIX, Farmingdale, NY) with pulse 1:1 s (on:off) in the presence or absence of 10 mM DTT. Samples at different time intervals (5, 10, 20 and 30 s) were withdrawn for phage morphology analysis by NS-TEM.

*Heat-induced disintegration:* Bacteriophage samples were incubated at 60 °C in the presence or absence of 10 mM DTT in a T-100 Thermal Cycler (BioRad). Samples at different time intervals (90, 180 and 300 s) were withdrawn for phage morphology analysis by NS-TEM.

### Negative-staining and transmission electron microscopy

A 3 microliter phage sample was applied to plasma-cleaned carbon film grids, stained by 2% uranyl acetate, and imaged on a Tecnai T12 electron microscope with standard settings. NS-TEM micrographs were used to analyze the intactness of phage particles after sonication or heating.

### Structural and sequence analysis

Structural and sequence similarity analysis were performed by DALI^37^ and NCBI-BLAST (https://blast.ncbi.nlm.nih.gov/Blast.cgi), respectively. Structural analysis was performed and displayed by ChimeraX^50^. The surface electrostatic potential was calculated by APBS—Adaptive Poisson-Boltzmann Solver^51^.

### Reporting summary

Further information on research design is available in the Nature Portfolio Reporting Summary linked to this article.

### Supplementary information

Supplemental Material

Reporting Summary

## Data Availability

The atomic models and three-dimensional reconstructions described in this paper are available in the Protein Data Bank (8FWB, 8FWC, 8FWE, 8FWG, 8FWM, 8FXR, 8FXP) and Electron Microscopy Data Bank (29500, 29501, 29503, 29504, 29512, 29541, 29540), respectively. All other data are available from the corresponding author on reasonable request.
