# Supplementary material for: Neck and capsid architecture of the robust Agrobacterium phage Milano
Source: Commun Biol. 2023 Sep 8;6:921. doi: 10.1038/s42003-023-05292-1 (PMC10491603; doi:10.1038/s42003-023-05292-1)
Supplement: Supplementary file 1 — Supplemental Material [file 42003_2023_5292_MOESM1_ESM.pdf]

Supplementary Materials

Neck and capsid architecture of the robust *Agrobacterium* phage Milano

Ravi R. Sonani[1], Nathaniel C. Esteves[2], Abigail A. Horton[2], Rebecca J. Kelly[2], Amanda L. Sebastian[2], Fengbin Wang[1,3], Mark A.B. Kreutzberger[1], Petr G. Leiman[4,*], Birgit E. Scharf[2,*], Edward H. Egelman[1,*]

[1]Department of Biochemistry and Molecular Genetics, University of Virginia School of Medicine, Charlottesville, VA 22903, USA

[2]Department of Biological Sciences, Virginia Tech, Blacksburg, VA 24061, USA

[3]Present address: Department of Biochemistry and Molecular Genetics, University of Alabama at Birmingham, Birmingham, AL 35233, USA

[4]Department of Biochemistry and Molecular Biology, University of Texas Medical Branch, Galveston, TX 77555, USA

This Supplement contains 11 figures and a table

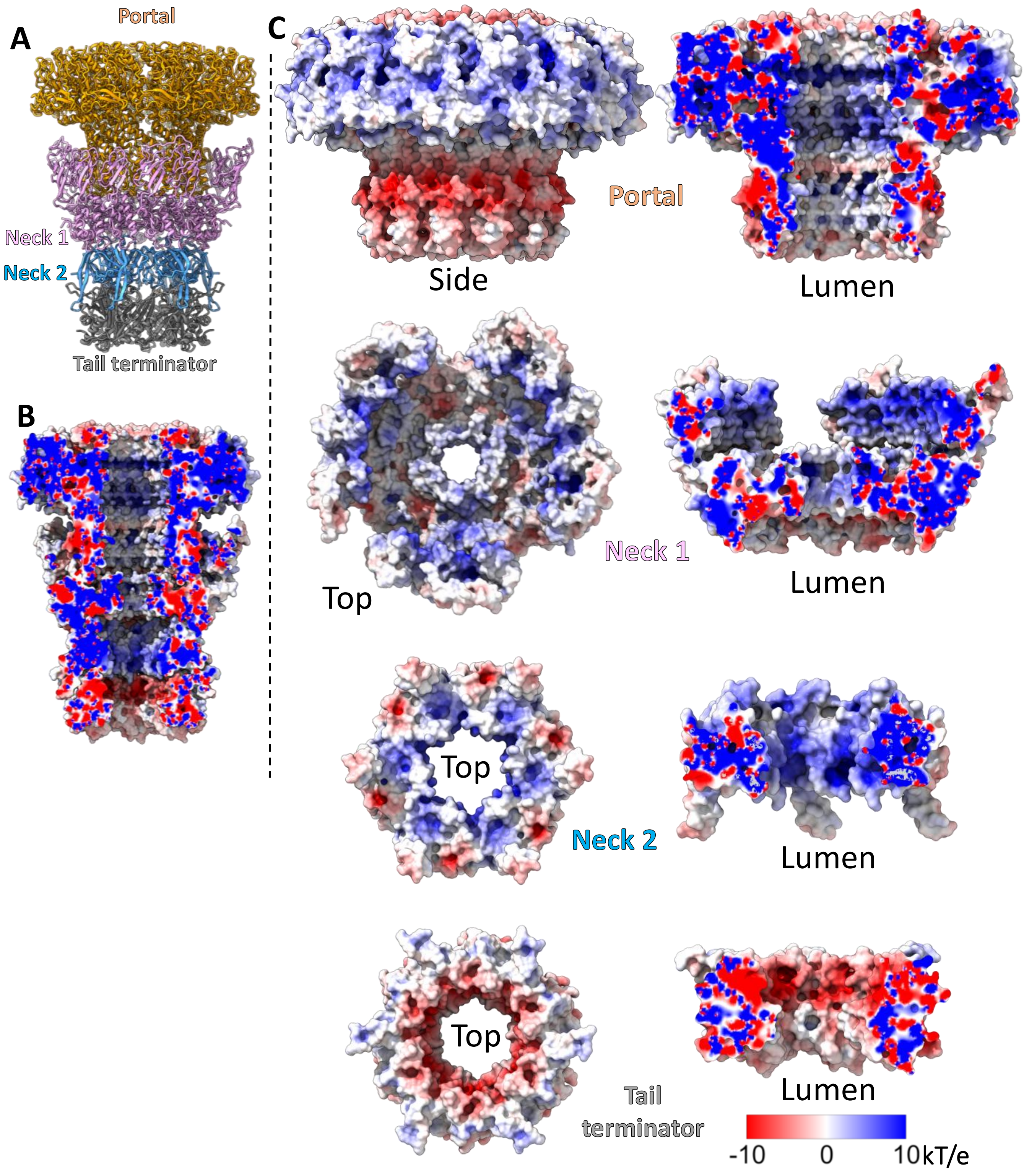

Supplementary Fig. S1: Electrostatic potential of the Milano neck tube. (A) The ribbon model of neck tube region (portal, neck 1, neck 2 and tail terminator), colored as in Fig. 1. (B) Electrostatic surface charges on the neck tube lumen. (C) Electrostatic surface charges of different rings: portal, neck 1, neck 2 and tail terminator rings of the Milano neck.

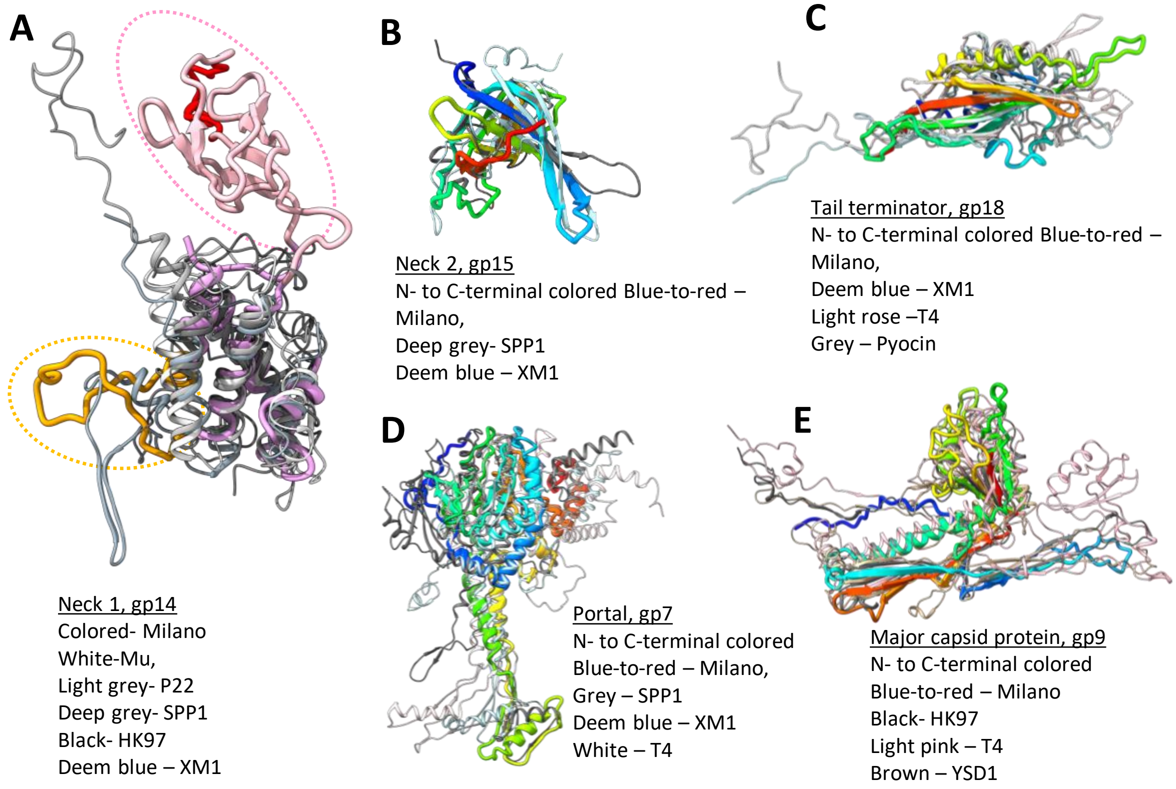

Supplementary Fig. S2: Superimposed structures of Milano neck proteins, (A) neck 1 protein, (B) neck 2 protein, (C) tail terminator protein, (D) portal protein and (E) MCP, with their structural homologues in other bacteriophages and pyocin. The coloring scheme for Milano neck 1 protein in (A) is the same as in Figure 2. In (B-E), the Milano protein is colored blue-to-red from N- to C-terminus.

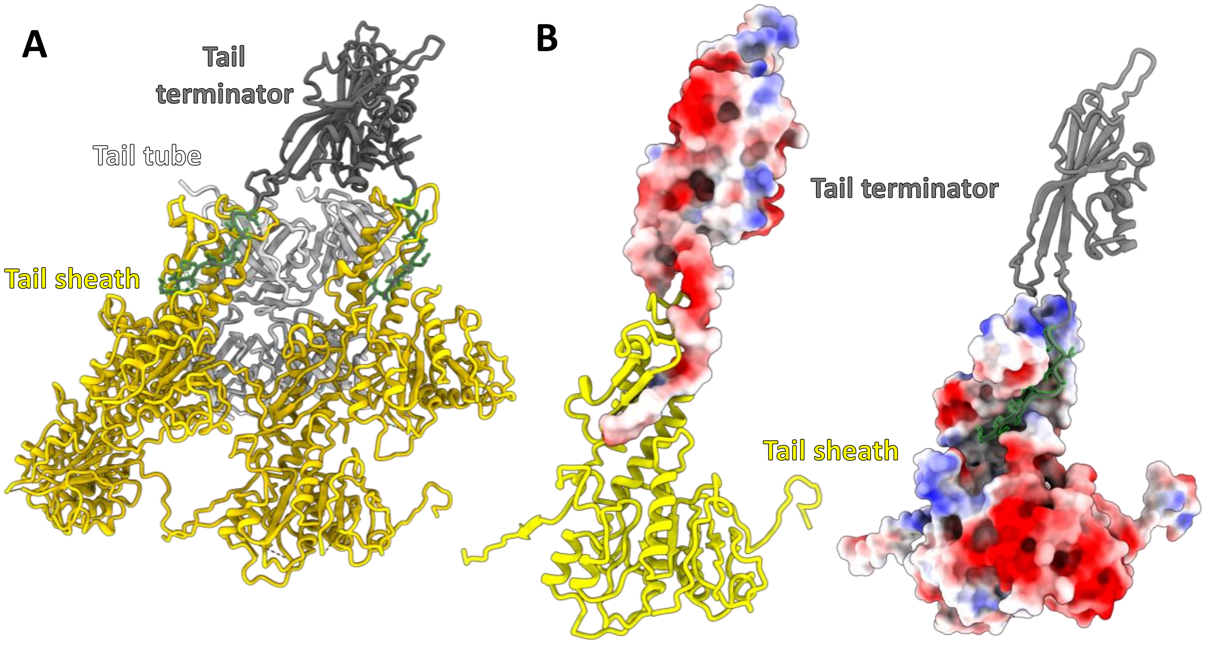

Supplementary Fig. S3: Interaction of tail terminator (gp18) with tail sheath (gp20). (A) The C-terminal strand (residues 164-175) of tail terminator burrows into the handshake domain of tail sheath subunits of the top ring. (B) Surface charges of the burrowing strand of tail terminator (left) and the handshake domain groove of tail sheath (right). The negatively charged strand of tail terminator burrows into the positively charged groove in tail sheath. The electrostic colr scale is as in Fig. S1.

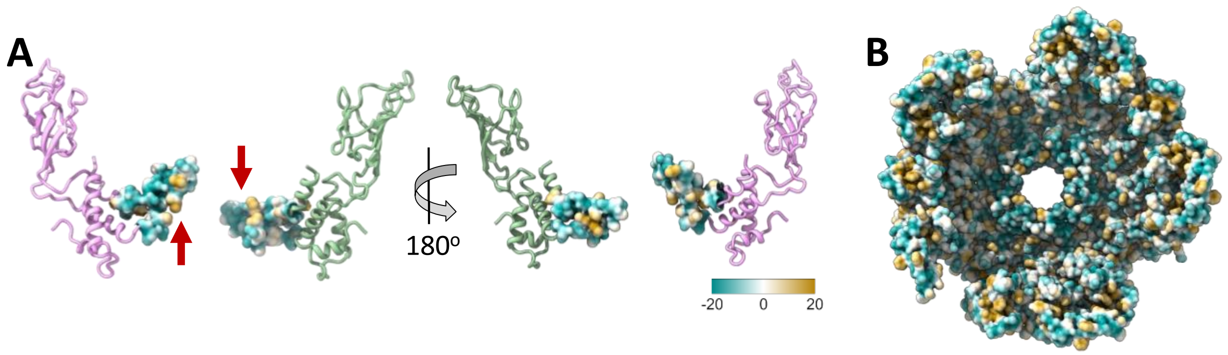

Supplementary Fig. S4: Lipophilic surface representation of stopper-loop of neck 1 protein (gp14) forming the genome gate, where cyan is polar and gold is hydrophobic (A) Side view of two protomers showing representative up and down conformations. (B) Top view of whole neck 1 showing the genome gate.

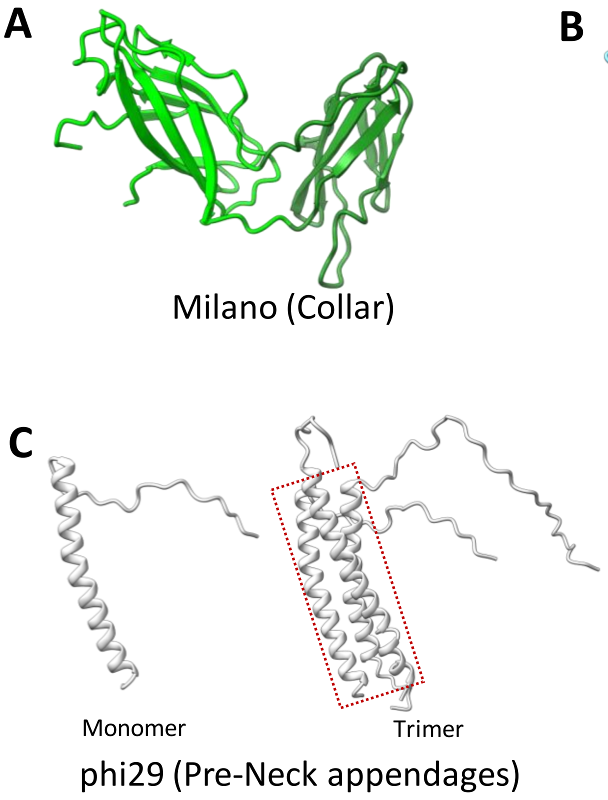

Supplementary Fig. S5: Milano collar lacks the typical triple-helical coiled-coil organization found in T4, phi29 and XM1 bacteriophage collar structures. The structure of (A) Milano collar protein (gp13) compared to (B) T4 collar protein (gp *wac*), (C) phi29 Pre-neck appendages (gp12) – equivalent to the collar and (D) XM1 collar protein (gp40). The typical triple-helical coil organization in T4, phi29 and XM1 collar structures are shown within red rectangles.

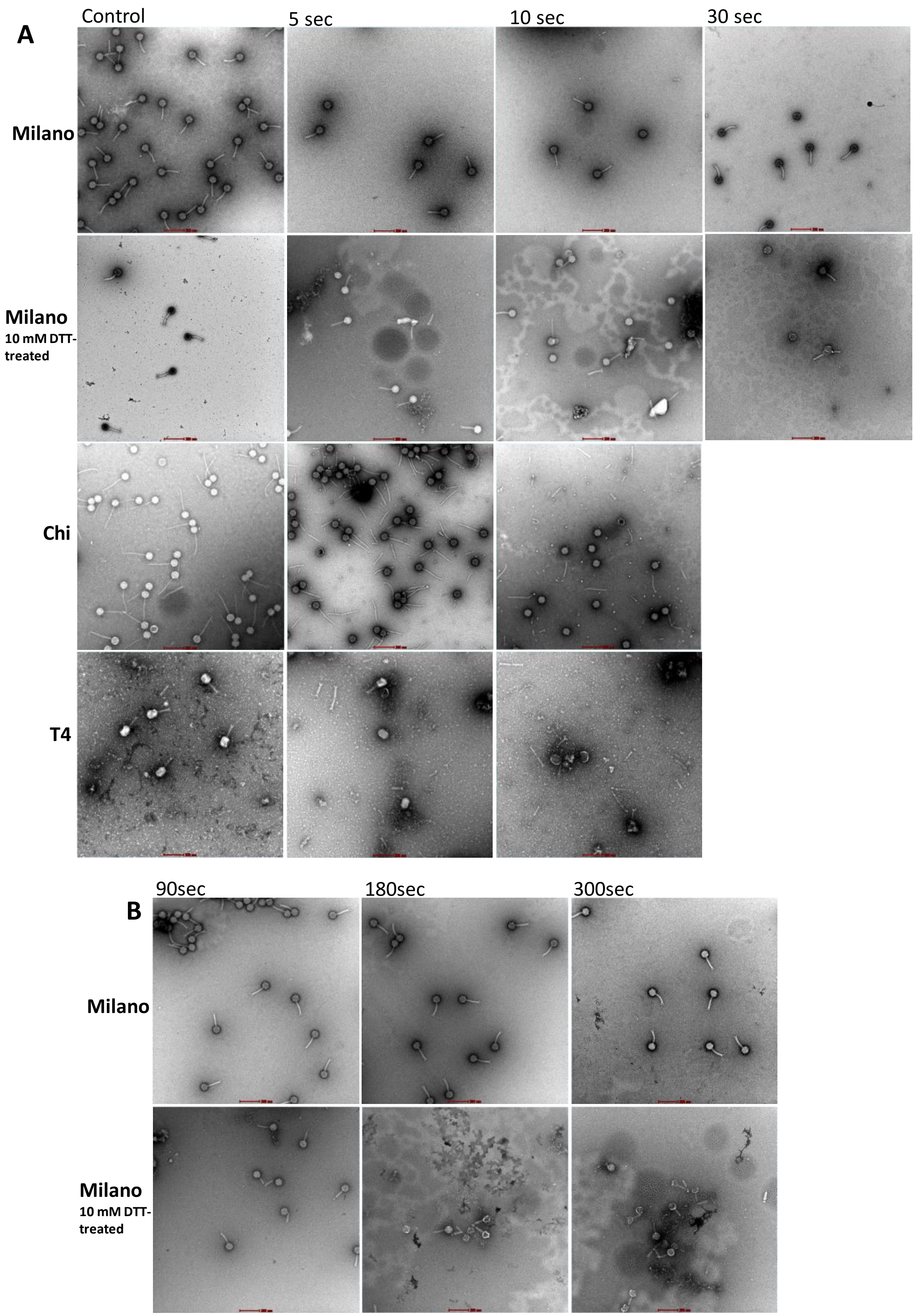

Supplementary Fig. S6: Phage disintegration assay. (A) NS-TEM images of disintegrated Milano, Chi and T4 bacteriophage particles by ultra-sonication. Milano particles stay intact for more than 30 sec of ultra-sonication, while the Chi, T4 and DTT-treated Milano particles start disintegrating after 5-10 sec. (B) NS-TEM images of heat-disintegrated Milano particles. Milano particles stay intact for more than 5 min during the heat treatment (60 ^{o}C). The DTT-treated Milano particles start disintegrating after 90 sec of heating. The sale bar is 200 nm.

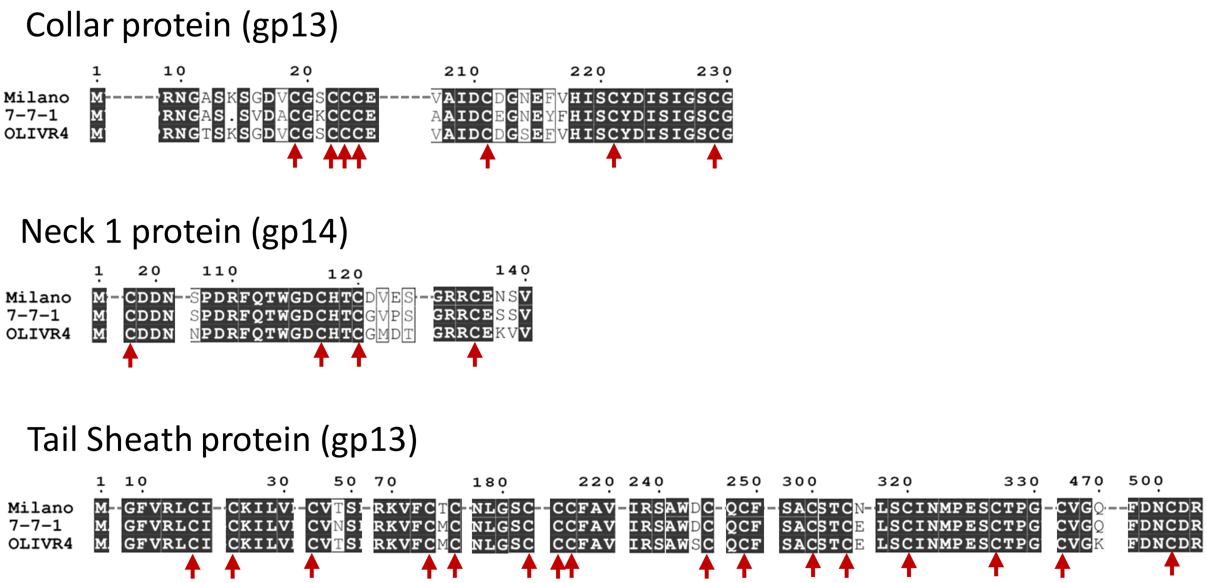

Supplementary Fig. S7: Sequence conservation in the collar, neck 1 and tail sheath proteins of *Agrobacterium* infecting phages: Milano, 7-7-1, and OLIVR4. Conserved cysteines are indicated by red arrows.

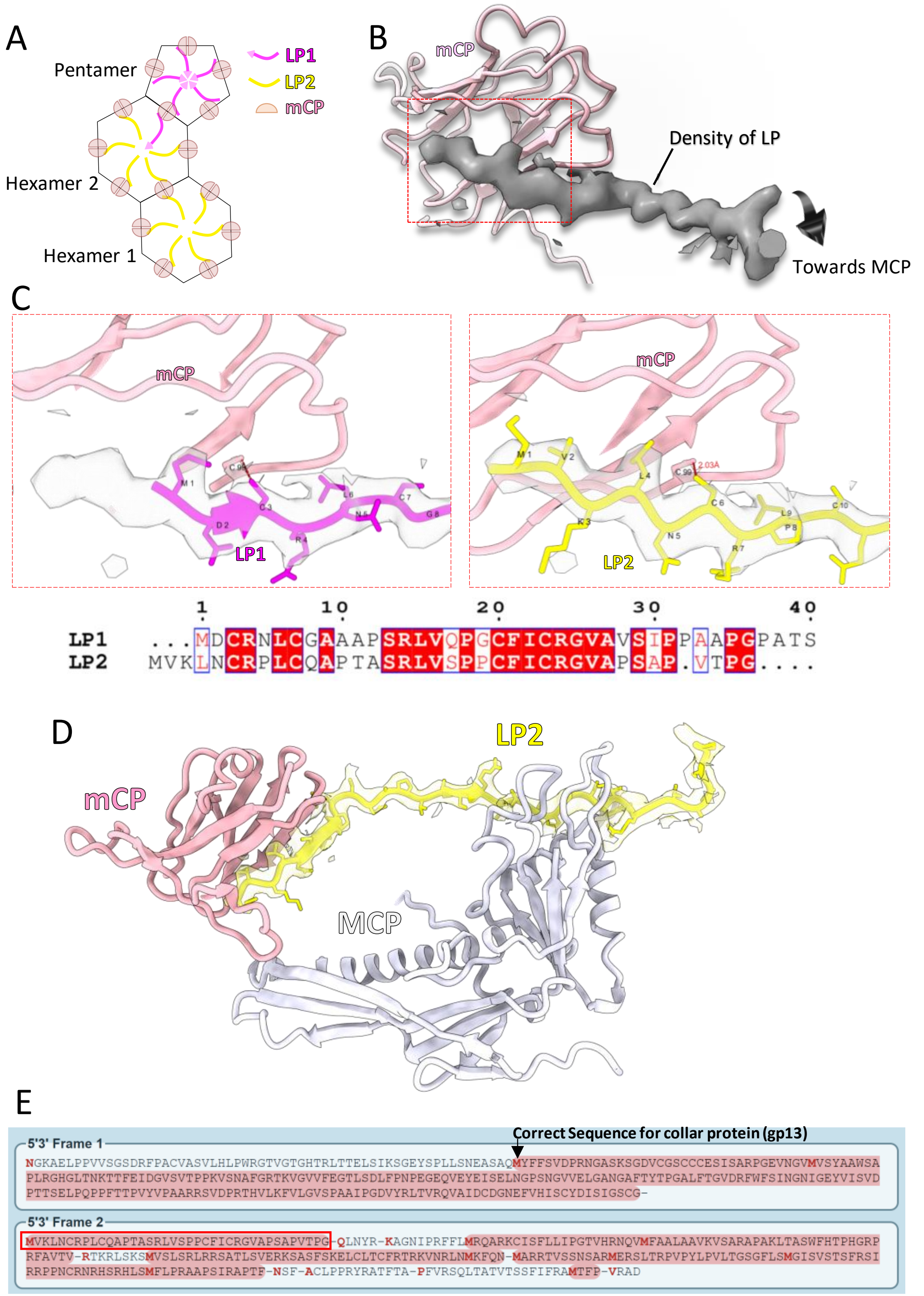

Supplementary Fig. S8: Cryo-EM map-based identification and fitting of LP2 in Milano capsid density map. (A) LP2 links minor capsid protein (mCP) to the major capsid protein (MCP) at 11 instances (yellow lines) out of total 17 in the asymmetric unit of the capsid. (B) Density corresponds to the N-terminal strand of linking protein interacting with mCP for ten LP2 instances shown in yellow in Fig. A. (C) The model fitting of LP1 (left) and LP2 (right) in the density shown in Fig. B. The lower panel presents a sequence alignment of LP1 and LP2. LP2 fits this density well due to its additional three residues compared to LP1. (D) Overall model showing LP2 linking the mCP to the MCP. (E) Open reading frames present in the reading frame 1 of the currently annotated gene of the collar protein (gp13). The density for the N-terminal 56 residues of gp16 (Uniport# A0A482MGH3) is not present in the relevant collar map, but instead appears as the separate protein - LP2 (red box: residues 1-38 in reading frame 2), for which the density has been identified in the capsid as shown in A-D. The collar open reading frame starts from Met-57 (in frame 1) and its previously annotated residues 1-56, in a reading frame 2, represents LP2 (gp128: red box: residues 1-38 in reading frame 2) followed by the small spacer.

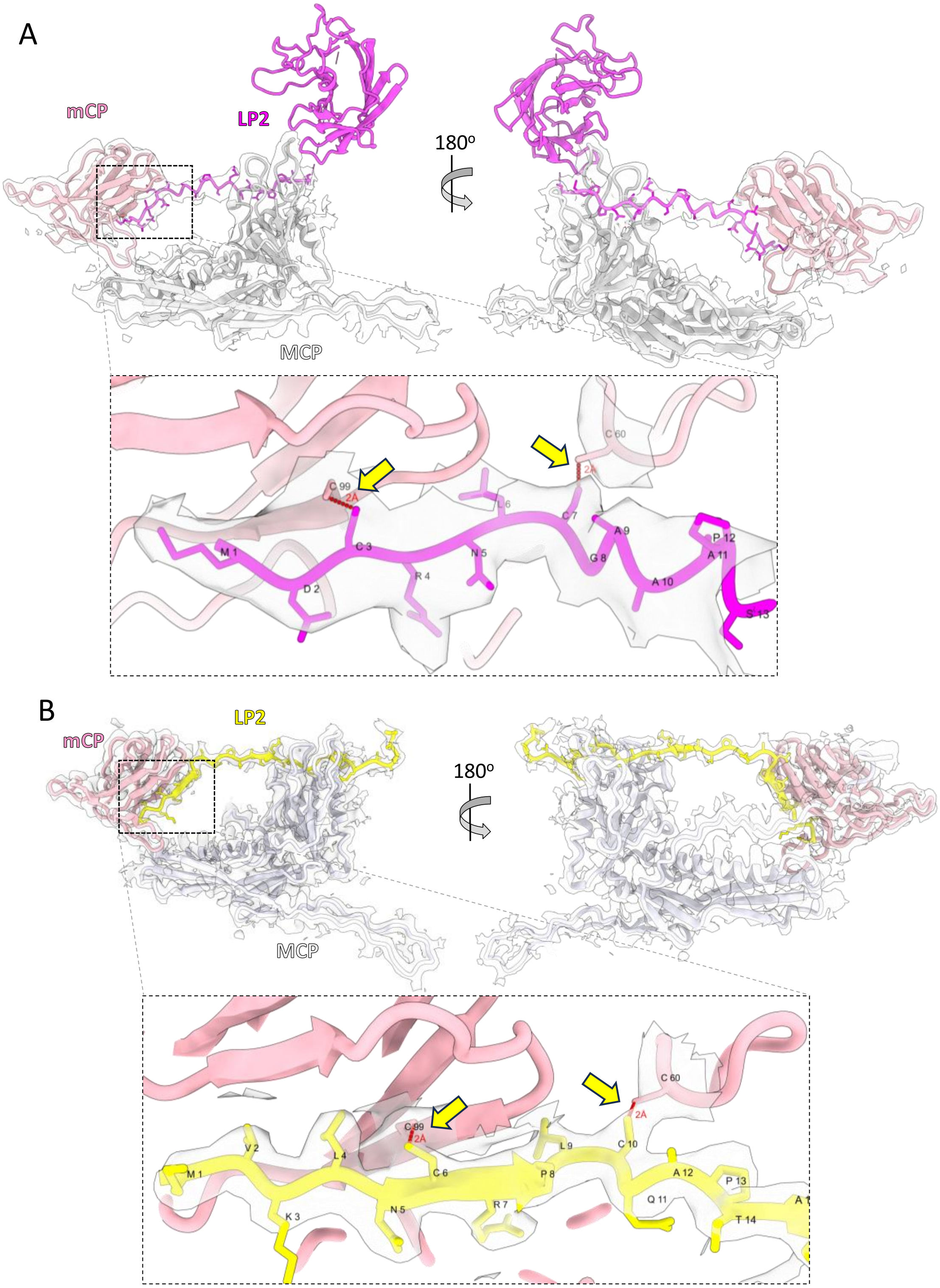

Supplementary Fig. S9: Linking protein 1 (LP1) **(A)** and 2 (LP2) **(B)** link the major capsid protein (MCP) to the minor capsid protein (mCP). Density corresponds to the LP1 β-sheet Ig-type domain is not resolved in the map thus the depicted model is AlphaFold predicted model. The quasi-equivalent interactions of the N-terminal strand of LP1 and LP2 with mCP are shown in the magnified view. LP1Cys3/LP2-Cys6 and LP1-Cys7/LP2-Cys10 are forming respective disulfide bonds with mCP-Cys99 and mCP-Cys60 as shown by yellow arrows.

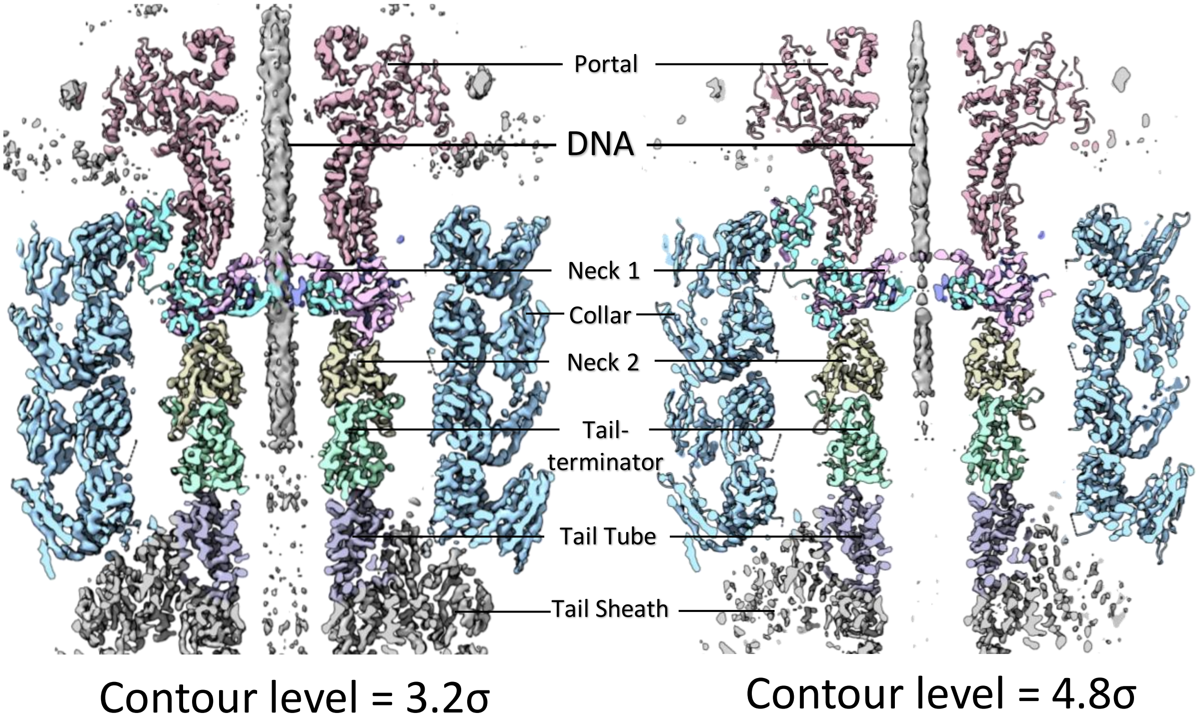

Supplementary Fig. S10: Density arising from the genomic DNA in the lumen of the Milano neck.

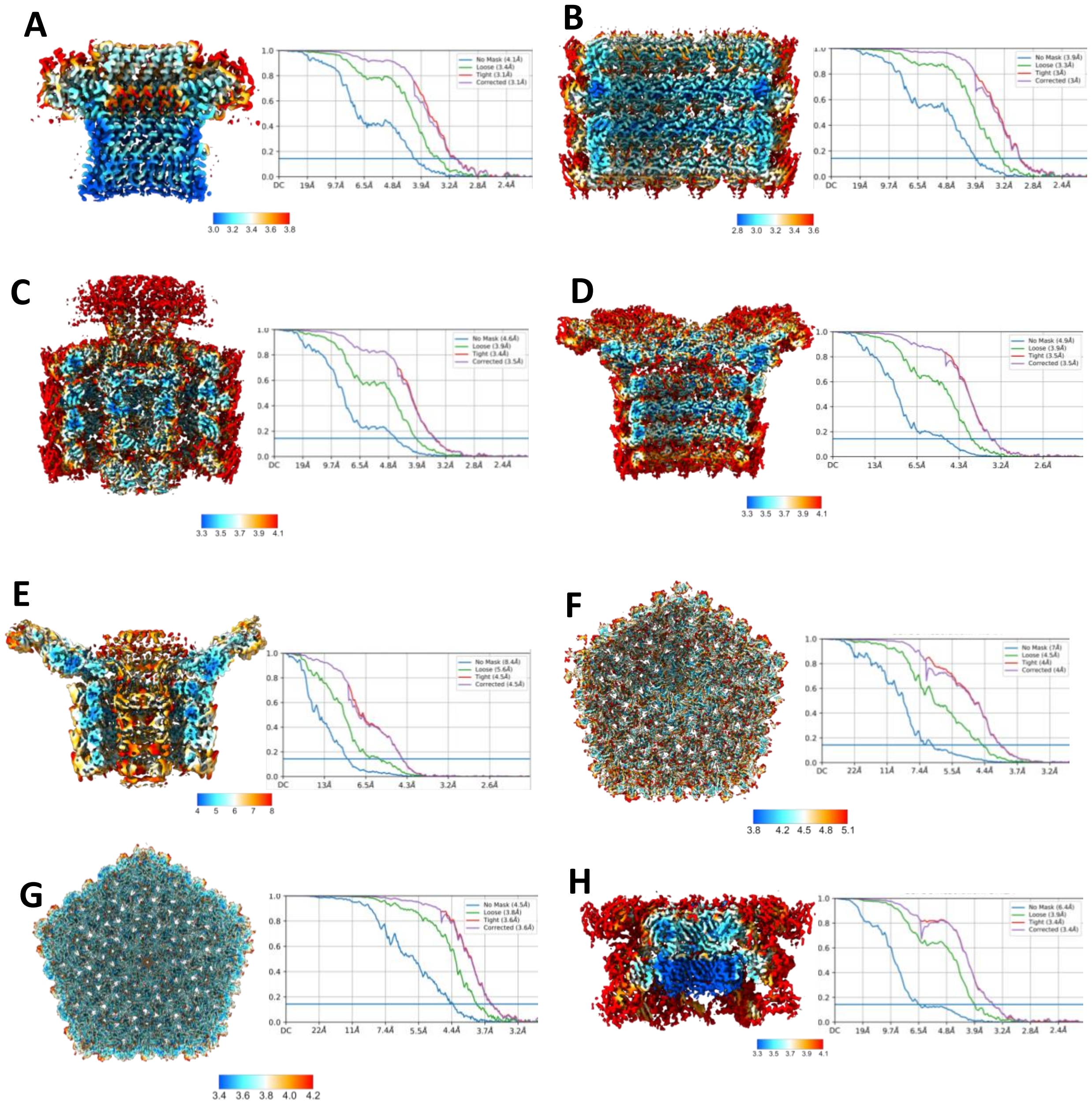

Supplementary Fig. S11: The local resolution map (left) and map:map FSC curves (right) with 0.143 cutoff for (A) 12-fold averaged portal reconstruction and (B) 15-fold averaged collar sheath reconstruction. For the neck region: (C) 3-fold averaged, (D) 5-fold averaged and (E) asymmetric reconstruction. (F) 5-fold and (G) icosahedrally-averaged capsid reconstruction. (H) 3-fold averaged focused reconstruction of neck-tail junction of Milano bacteriophage. The map shown in (E) is locally filtered to remove high frequency noise.

Supplementary Table S1: DALI structural similarities search hits for collar protein (gp13), linking protein 1 (LP1, gp16) and mCP (gp10) of Milano. Glycan/lipid/receptor/cell-surface binding proteins and adhesive proteins are highlighted in grey.

	No:	Chain	Z	rmsd	lali	nres	%id	Description
Collar protein (gp13)	1:	4m00-A	7.8	13.3	85	501	14	SERINE-RICH ADHESIN FOR PLATELETS
	2:	6dlh-A	7.8	12.7	106	703	15	ALPHA-1,4-ENDOFUCOIDANASE
	3:	2yhg-A	7.8	2.7	95	411	11	CELLULOSE-BINDING PROTEIN
	4:	4nzj-A	7.7	8.9	97	468	8	PUTATIVE ALPHA-GALACTOSIDASE
	5:	6s5w-A	7.6	2.8	83	106	17	SURFACE PROTEIN
	6:	1eut-A	7.3	2.6	94	601	14	SIALIDASE
	7:	4uj6-A	7.3	13.3	91	711	7	SURFACE LAYER PROTEIN
	8:	4ak1-A	6.8	21.7	121	600	9	BT_4661: SURFACE POLYSACCHARIDE BINDING PROTEIN
	9:	3pnr-B	6.8	3.4	93	129	8	FALCIPAIN 2
	10:	8dke-P	6.8	2.5	85	333	14	ISOFORM 2 OF CYSTINOSIN
	11:	6tct-A	6.7	2.8	90	125	12	MAKD
	12:	5ngj-A	6.7	7.9	93	440	12	TAIL TUBE PROTEIN
	13:	7djl-A	6.7	3.8	93	473	10	PROTEIN SUPPRESSOR OF QUENCHING 1
	14:	2j2z-A	6.7	2.8	91	216	9	CHAPERONE PROTEIN PAPD
	15:	6gyb-B	6.7	2.4	80	223	13	VIRB7
	16:	4aq1-A	6.6	10.8	96	721	6	SBSB PROTEIN
	17:	4wiq-A	6.5	3.3	84	231	18	DYSTROGLYCAN
	18:	6qx4-B	6.5	2.7	82	593	9	S-LAYER PROTEIN SAP
	19:	7z4f-G	6.5	29.4	107	922	11	PUTATIVE STRUCTURAL PROTEIN
	20:	5n5p-A	6.5	2.9	95	138	16	PUTATIVE CELLULOSOMAL SCAFFOLDIN PROTEIN
Linking protein 1 (gp16)	1:	7ofq-b	5.6	4.5	121	213	7	ARCHAELLIN
	2:	1bwm-A	5.1	4.2	108	249	6	PROTEIN (ALPHA-BETA T CELL RECEPTOR (TCR) (D10))
	3:	4a2m-B	5	3.6	107	750	5	TWO-COMPONENT SYSTEM SENSOR HISTIDINE KINASE
	4:	3vmn-A	5	2.8	88	633	9	DEXTRANASE
	5:	5j67-C	4.9	7.6	115	563	10	ASTROTACTIN-2
	6:	2yn3-C	4.8	4	110	284	13	PUTATIVE INNER MEMBRANE PROTEIN
	7:	3va6-A	4.8	3.9	105	747	8	TWO-COMPONENT SYSTEM SENSOR HISTIDINE KINASE
	8:	6pal-A	4.7	8.8	99	404	8	UNCHARACTERIZED PROTEIN
	9:	4ow5-A	4.7	3.1	96	307	5	FUSOLIN
	10:	7lhk-B	4.7	4	97	298	5	NUCLEAR ELONGATION AND DEFORMATION PROTEIN
	11:	1doa-B	4.7	7.1	114	200	5	PROTEIN (GTP-BINDING PROTEIN)
	12:	3hlk-B	4.6	5.3	100	411	6	ACYL-COENZYME A THIOESTERASE 2
	13:	5f0j-B	4.6	4	107	306	7	VACUOLAR PROTEIN SORTING-ASSOCIATED PROTEIN 35
	14:	2xwx-A	4.5	6.4	101	385	4	GLCNAC-BINDING PROTEIN A
	15:	5hzv-A	4.4	4	106	598	8	MALTOSE-BINDING PERIPLASMIC PROTEIN
	16:	5a42-A	4.4	7.5	99	1597	11	UNCHARACTERIZED LIPOPROTEIN YFHM
	17:	3dgc-S	4.4	6.8	92	207	8	INTERLEUKIN-22 RECEPTOR
	18:	2pn5-A	4.4	4	99	1283	10	THIOESTER-CONTAINING PROTEIN I
	19:	7eeb-F	4.4	3.6	91	1042	8	ENHANCED GREEN FLUORESCENT PROTEIN
	20:	4hjj-H	4.4	3.5	96	341	11	ANTI-IL12 ANTI-IL18 DFAB HEAVY CHAIN
	21:	1f13-A	4.4	4.4	101	721	11	CELLULAR COAGULATION FACTOR XIII ZYMOGEN
	22:	5tfy-A	4.4	3.7	95	164	13	FLAGELLIN
	23:	4zel-A	4.3	3.2	100	550	10	DOPAMINE BETA-HYDROXYLASE
	24:	6tn6-A	4.3	3.7	99	465	13	MANNAN ENDO-1

	25:	8ema-A	4.3	3.5	98	597	8	ISOFORM 2 OF IMMUNOGLOBULIN HEAVY CONSTANT MU
minor Capsid Protein (gp10)	1:	6i56-A	8.4	4.4	94	279	12	PHAGE-LIKE ELEMENT PBSX PROTEIN XEPA
	2:	4e0s-A	6.3	3.2	90	1552	7	COMPLEMENT C5
	3:	7kve-B	6.2	5.2	105	1374	6	COAGULATION FACTOR V
	4:	5awo-A	6.1	3.6	100	596	9	ISOMALTODEXTRANASE
	5:	7k66-A	6.1	3.2	99	1265	10	COAGULATION FACTOR VIII
	6:	3j4u-H	6.1	2.9	91	140	11	MAJOR CAPSID PROTEIN
	7:	3afg-B	5.8	3.6	97	507	12	SUBTILISIN-LIKE SERINE PROTEASE
	8:	6ds5-A	5.8	3	92	160	5	SEIPIN
	9:	4ag4-A	5.6	3.1	99	340	6	EPITHELIAL DISCOIDIN DOMAIN-CONTAINING RECEPTOR 1
	10:	4gz9-A	5.6	3.6	102	562	10	NEUROPILIN-1
	11:	2pn5-A	5.5	3.5	88	1283	8	THIOESTER-CONTAINING PROTEIN I
	12:	4deq-A	5.5	3.1	96	217	17	NEUROPILIN-1
	13:	3nqh-A	5.5	2.8	86	439	7	GLYCOSYL HYDROLASE
	14:	1pnf-A	5.4	3.4	101	314	8	DI-N-ACETYLCHITOBIOSE
	15:	5i5k-B	5.3	3.3	92	1634	8	COMPLEMENT C5
	16:	6wpx-B	5.3	3.5	92	451	10	BLEST2
	17:	8fdg-A	5.3	4.5	104	1505	5	COAGULATION FACTOR V
	18:	2v5d-A	5.3	3.9	97	722	7	O-GLCNACASE NAGJ
	19:	1nlq-E	5.2	3.6	88	98	7	NUCLEOPLASMIN-LIKE PROTEIN
	20:	7zzz-A	5.2	3.4	87	239	8	MAJOR CAPSID PROTEIN P5
	21:	5w6h-A	5.1	2.9	93	697	14	TAILSPIKE PROTEIN 4
	22:	6s44-A	5.1	3.1	90	146	6	CAPSID PROTEIN
	23:	4xup-A	5.1	3.7	101	330	11	ENDO-1
	24:	3j31-Q	5.1	3.2	90	220	2	A223 PENTON BASE
	25:	6a48-A	5.1	4.2	101	651	7	REELIN
